# Functional biopolymeric materials for active food packaging: the case of monolayer films of thermoplastic starch and olive leaf extract

**DOI:** 10.3389/fbioe.2025.1672740

**Published:** 2025-10-22

**Authors:** Rita Sousa, José M. Silva, Lidia Verano-Naranjo, Cristina Cejudo-Bastante, William M. Facchinatto, Adelaide Almeida, Armando J. D. Silvestre, Carmen S. R. Freire, Carla Vilela

**Affiliations:** ^1^ CICECO – Aveiro Institute of Materials, Department of Chemistry, University of Aveiro, Aveiro, Portugal; ^2^ Chemical Engineering and Food Technology Department, Faculty of Science, Wine and Agrifood Research Institute (IVAGRO), University of Cadiz, Puerto Real, Spain; ^3^ CESAM – Centre for Environmental and Marine Studies, Department of Biology, University of Aveiro, Aveiro, Portugal

**Keywords:** thermoplastic starch, olive leaf extract, biobased films, antioxidant activity, antibacterial action, food shelf-life, active packaging

## Abstract

Active packaging materials based on biopolymers and natural additives represent a significant innovation, offering a sustainable solution of packaging combined with multifunctional bioactive properties to extend the shelf-life of food products. In the present study, bioactive films composed of thermoplastic starch (TPS) and an olive leaf extract (OLE) were fabricated via solvent casting. OLE was used in different concentrations, namely 5 and 10 wt.%, and the films were plasticized with glycerol (20 wt.%). The ensuing homogeneous films are translucent (50% < transmittance <80%, opacity <2.3 mm^−1^) and exhibited a light-green coloration characteristic of OLE. The inclusion of OLE had a positive effect on the mechanical performance (Young’s modulus ≈1.1 GPa), water resistance (water solubility <29% and moisture absorption ≤45%) and thermal stability (up to 200 °C) of the films. Furthermore, the incorporation of OLE in the plasticized TPS films originated materials with UV-blocking properties (transmittance ≤28%) and high antioxidant activity (DPPH scavenging activities of ca. 90%), as well as antibacterial action against a methicillin-resistant *Staphylococcus aureus* bacterial strain (maximum reduction of ca. 2.6–log [colony-forming units (CFU) mL^–1^] after 72 h). Lastly, when used to pack sliced pears stored at +4 °C for 7 days, the TPS/OLE-based films effectively delayed browning, mass loss and pH changes. Overall, the incorporation of OLE into plasticized TPS films demonstrates great potential for sustainable active food packaging, offering an approach to preserve the quality and extend the shelf-life of fresh food products.

## 1 Introduction

Food packaging is essential to protect and extend the shelf-life of food products. Nevertheless, the cumulative use of disposable fossil-based synthetic plastics has significant environmental impacts, not only due to their slow degradation but also because of the substantial environmental costs of their production, including intensive use of primary energy and a high carbon footprint (Ncube et al., 2021; Rosenboom et al., 2022). With the increase in environmental awareness, there has been a growing interest in the search for sustainable and biodegradable alternatives (Guillard et al., 2018; Rosenboom et al., 2022; Kim et al., 2023). Among the most promising candidates are natural polymers (Abdulla et al., 2024), such as polysaccharides (e.g., starch (Almeida et al., 2023), cellulose (Bastante et al., 2021), chitosan (Vilela et al., 2017; Khan et al., 2024b), agar (Makhloufi et al., 2021; 2022), curdlan and alginate (Khan et al., 2024a)) and proteins (e.g., collagen (Marangoni Júnior et al., 2021) and gelatin (Sun et al., 2024)).

Starch, in particular, is widely available, renewable, cost-effective, and compostable polysaccharide, making it an attractive polymeric substrate for the development of biodegradable packaging films (Khan et al., 2017). Native starch, a polysaccharide composed of amylose (long chains of glucose units linked by α-1,4 glycosidic bonds) and amylopectin (long chains of glucose units linked by α-1,4 and α-1,6 glycosidic bonds), can be transformed into thermoplastic starch (TPS) by applying heat and shear force in the presence of sufficient water (Nafchi et al., 2013). TPS can be processed using conventional industrial techniques, such as extrusion and injection molding, making it a promising alternative to traditional fossil-based thermoplastics (Khan et al., 2017; Surendren et al., 2022). However, TPS suffers from poor mechanical strength, limited flexibility and high moisture sensitivity, which hinders its direct and broader application (Khan et al., 2017; Surendren et al., 2022). To address these limitations, starch can be chemically modified (Masina et al., 2017), blended with other biopolymers to improve specific properties (Surendren et al., 2022), or combined with natural or synthetic additives (Muñoz-Gimena et al., 2023) like nanocellulose fibers for improved mechanical performance (Almeida et al., 2023), glycerol or sorbitol for improved flexibility and processability (Jha, 2020) and reduced graphene oxide for reduction of moisture sensitivity (Alves et al., 2022).

Despite these enhancements, TPS-based films still require functional improvements to meet the growing demand for active food packaging systems. Active packaging goes beyond traditional passive protection by incorporating components that can interact with the food or its environment to extend shelf-life and ensure safety (Vilela et al., 2018; Carvalho et al., 2021). One approach for achieving active functionality is the incorporation of natural bioactive agents with antioxidant and antibacterial properties, such as essential oils (Vianna et al., 2021) and plant derived extracts (Zhu, 2025), and thus providing environmentally friendly alternatives. While several natural extracts rich in polyphenolic compounds, including rosemary extract (Piñeros-Hernandez et al., 2017), green tea leaf extract (Milovanovic et al., 2024), turnip peel extract (Khaledian et al., 2024), Hom Nil rice extract (Sarak et al., 2024) and olive fruit extract (García et al., 2020), have been explored in TPS formulations, there has been no prior research focusing on the specific combination between thermoplastic starch and olive leaf extract (OLE).

OLE is a promising bioactive additive due to its high content of phenolic compounds, particularly oleuropein, hydroxytyrosol, and verbascoside, which have demonstrated strong antioxidant activity and antibacterial effect towards common foodborne pathogens (Grabska-Zielińska, 2024). Olive leaves are an abundant by-product of olive oil production particularly in the Mediterranean area, and their valorization aligns with the principles of agricultural waste reuse and circular economy. These properties make OLE a viable additive for active packaging materials aimed at improving food preservation and safety (Grabska-Zielińska, 2024). Notably, OLE has been loaded into both non-biodegradable and biodegradable polymers from synthetic or biobased origin via conventional or innovative and eco-friendly film-processing methodologies. For example, OLE was incorporated into a non-biodegradable food-grade poly(ethylene terephthalate)/polypropylene (PET/PP) multilayer film, effectively delaying the lipid oxidation of sunflower seeds during 15 days of storage (Cejudo Bastante et al., 2018) and extending the shelf-life of cherry tomatoes by 20 days (Cejudo Bastante et al., 2019a). In another study, the inclusion of OLE into a commercial biodegradable ternary film of poly(lactic acid)/poly(butylene adipate-*co*-terephthalate)/thermoplastic starch (PLA/PBAT/TPS) reduced the chilling injury symptoms of green peppers during 20 days of cold storage at +4 °C (Verano-Naranjo et al., 2025). Furthermore, biodegradable OLE-loaded carrageenan films were able to efficiently slow the growth of psychrophilic microorganisms in packed lamb meat (Martiny et al., 2020b), whereas OLE-loaded biodegradable chitosan films could be a solution for wrapping meat hamburgers, as they helped reduce microbial spoilage during 20 days of storage (Famiglietti et al., 2022).

To the best of our knowledge, the combination of TPS and OLE to develop monolayered films for packaging fresh-cut fruits has not yet been investigated. This gap is particularly relevant given the importance in valorizing agricultural by-products (Grabska-Zielińska, 2024), along with OLE’s rich polyphenolic profile and concomitant potential to improve the functional properties of TPS-based films.

In this context, the aim of the present study is to fabricate bioactive films composed of thermoplastic starch and olive leaf extract for application as sustainable active food packaging materials. Films with different concentrations of OLE (obtained via enhanced solvent extraction (ESE)) were plasticized with glycerol and were characterized in terms of structure, morphology, thermal stability, mechanical performance, optical properties, water barrier properties, antioxidant activity, antibacterial activity against a methicillin-resistant *Staphylococcus aureus* (MRSA) strain and applicability in preserving minimally processed fresh-cut fruits during cold storage.

## 2 Materials and methods

### 2.1 Chemicals, materials and microorganisms

Glycerol (≥99.5%), 2,2-diphenyl-1-picrylhydrazyl (DPPH) radical, phosphate buffer saline (PBS, pH 7.4) and sodium hypochlorite solution (NaClO, 6%–14% active chlorine, pH 12–13 (20 °C in H_2_O)) were acquired from Sigma-Aldrich (Lisboa, Portugal). Tryptic soy agar (TSA) and tryptic soy broth (TSB) were supplied by Liofilchem (Roseto degli Abruzzi TE, Italy). Ultrapure water (type 1) with a resistivity of 18.2 MΩ cm and conductivity of 0.056 μS cm^–1^ at 25 °C was obtained with a Simplicity^®^ Water Purification System (Merck, Darmstadt, Germany). Other chemicals and solvents were of laboratory grade.

Potato starch (white powder) with 20% amylose and 80% amylopectin, residue on ignition (ash) ≤ 0.4%, molecular mass of 342.30 gmol^–1^, pH of 5.0–7.0 (at 25 °C and 2% in solution), viscosity of ∼300 cP (at 25 °C and 2% in solution), density of 0.14 g cm^–3^, and melting point of 256 °C–258 °C, was purchased from Sigma-Aldrich (Lisboa, Portugal). Food-grade poly(vinyl chloride) (PVC) cling film with a thickness of 16 ± 1 μm, branded with a white label, was acquired from a retail supermarket.

The olive leaves from the *Olea europaea* Hojiblanca olive cultivar were provided by the associated olive oil mills of San José Lora de Estepa (Sevilla, Spain). The leaves were dried in an air-ventilated oven (Thermo Fisher Scientific, Massachusetts, United States) at 40 °C for 48 h, and crushed with an electric grinder (Moulinex A980, France).

Pears (*Pyrus communis* L. cv. Rocha), grown in Portugal, were purchased at a local market in Aveiro (Portugal). Fruits with a diameter between 55 and 60 mm, were chosen according to color uniformity and absence of physical defects.

Methicillin-resistant *S*. *aureus* (MRSA) DSM 25693 bacterium was provided by DSMZ–Deutsche Sammlung von Mikroorganismen und Zellkulturen GmbH (German Collection of Microorganisms and Cell Cultures).

### 2.2 Preparation of the olive leaf extract (OLE)

Enhanced solvent extraction (ESE) in batch mode was used to obtain OLE using a supercritical extraction equipment, model SF1000, provided by Thar Technologies (Pittsburgh, PA, United States). The extraction conditions were previously reported (Verano-Naranjo et al., 2025). Briefly, 120 g of dried and ground olive leaves were introduced into the 1,000 mL extraction vessel, together with 600 mL of ethanol. Then, the vessel was heated until 80 °C and the CO_2_ was pumped at 10 g min^–1^ until reaching 120 bar. After 12 h under static conditions, the system was depressurized and left to cool, and the OLE was recovered from the vessel. The resulting ethanolic extract with a concentration of 67.4 ± 1.6 g L^–1^ was stored at +4 °C. The two primary compounds identified in the extract were oleuropein and luteolin 7-O-glucoside, with concentrations analogous to those reported by Verano-Naranjo et al. (2025).

### 2.3 Preparation of the TPS-based films

The films were prepared by solvent casting according to a method described in literature with some modifications (Almeida et al., 2023). Starch (2% (w/v)) was dispersed in distilled water and the obtained suspension was heated in an oil bath at 95 °C for 30 min with stirring at 500 rpm to promote the gelatinization of starch. After this step, the starch solution was cooled down to approximately 50 °C, followed by the addition of glycerol (20 wt.%, on starch dry basis), and the solution stirred for another 1 h. The TPS/OLE films were prepared by adding two different concentrations of OLE, namely 5 and 10 wt.% (relative to starch), to the starch solution (Table 1). All formulations were casted in polystyrene Petri dishes and placed in an air-ventilated oven at 40 °C for 24 h for solvent evaporation. After drying, the films were carefully peeled off from the Petri dishes and stored at room temperature in a desiccator.

**TABLE 1:** Identification, composition, and thickness of the prepared films (different letters above the thickness values correspond to statistically significant differences).

Film	Starch (%, w/v)	Glycerol (wt.%)	OLE (wt.%)	Thickness (µm)
TPS	2.0	20.0	0	69 ± 7^a^
TPS_OLE5	2.0	20.0	5.0	72 ± 3^a^
TPS_OLE10	2.0	20.0	10.0	76 ± 3^a^

### 2.4 Characterization methods

#### 2.4.1 Thickness

The thickness of the films was measured with a hand-held digital micrometer MDC-25PX (Mitutoyo Corporation, Tokyo, Japan) with an accuracy of 1 μm. All measurements were randomly performed at seven different places on the films.

#### 2.4.2 Attenuated total reflection-fourier transform infrared (ATR-FTIR) spectroscopy

ATR-FTIR spectra were recorded with a Perkin-Elmer FT-IR System Spectrum BX spectrophotometer (Perkin-Elmer Inc., Massachusetts, United States) equipped with a single horizontal Golden Gate diamond ATR crystal cell, over the range of 500–4,000 cm^−1^ at a resolution of 4 cm^−1^ over 32 scans.

#### 2.4.3 X-ray diffraction (XRD)

XRD was performed on a Phillips X’pert MPD diffractometer (PANalytical, Eindhoven, Netherlands) using Cu Kα radiation (λ = 1.541 Å) with a scan rate of 0.05° s^−1^. The XRD patterns were collected in reflection mode with the test specimens (flat discs with 1.5 cm^2^) placed on a Si wafer (negligible background signal) for mechanical support.

#### 2.4.4 Scanning electron microscopy (SEM)

SEM micrographs were obtained with a HR-FESEM SU-70 Hitachi microscope (Hitachi High-Technologies Corporation, Tokyo, Japan) operating at 4 kV. The samples were placed on an aluminum plate and previously coated with a carbon film.

#### 2.4.5 Thermogravimetric analysis (TGA)

TGA was performed with a SETSYS Setaram TGA analyzer (SETARAM Instrumentation, Lyon, France) equipped with a platinum cell. The test specimens were heated from room temperature to 800 °C, at a constant rate of 10 °C min^−1^ under inert (N_2_) atmosphere.

#### 2.4.6 Tensile tests

Tensile properties were measured using an Instron 5966 Series equipment (Instron Corporation, United States) at room temperature, applying a velocity of 10 mm min^−1^ and gauge length of 30 mm, and using a static load cell of 500 N. Six specimens of 5.0 × 1.0 cm^2^ were measured for each film sample, and the average value was considered. The Instron BlueHill 3 software was used for determining the Young’s modulus, tensile strength, and elongation at break data.

#### 2.4.7 Optical properties

The colorimetric coordinates (CIELab scale: L*, a*, b*) and ISO brightness (ISO 2470–1) were measured with a Konica Minolta CM-2300d portable sphere type spectrophotometer (Konica Minolta Sensing Europe BV, United Kingdom) using a white calibration tile surface (Pereira et al., 2022; Matos et al., 2023). The parameters of lightness, L* (black (0) to white (100)) and color coordinates, a* (green = –a* to red = +a*) and b* (blue = –b* to yellow = +b*) were measured using the Spectra Magic™ NX software. All measurements were randomly performed at six different places of the film samples.

The transmittance of the samples was collected with a Shimadzu UV1800 UV–vis spectrophotometer (Shimadzu Corporation, Kyoto, Japan). Spectra were acquired at room temperature in the wavelength range from 200 to 700 nm in steps of 1 nm. Additionally, the opacity of the films was determined at 600 nm by the following equation (Almeida et al., 2023):

$$Opacity=\frac{A_{600}}{x}$$

where *A*_600_ is the absorbance at 600 nm and x is the mean thickness (mm) of the film. Measurements were performed in triplicate and the mean value with the respective standard deviation were calculated.

#### 2.4.8 Water solubility

The water solubility of the films, was assessed following a previous described method (Núñez-Flores et al., 2012). Briefly, film samples (2.0 × 2.0 cm^2^) were dried (60 °C, 4 h), weighed and placed in flasks with 25 mL of distilled water and then let to stand at room temperature for 24 h. Afterwards, the films were recovered in a filter paper and dried at 105 °C for 24 h. The solubility of each film was calculated by the equation:

$$Solubility\;in\;water\;\left(\%\right)=\frac{W_{0}-W_{f}}{W_{0}}\times 100$$

where *W*_*0*_ is the initial mass of the films and *W*_*f*_ is the mass of the undissolved dried film fraction. Measurements were carried out in five replicates.

#### 2.4.9 Moisture absorption capacity

The moisture absorption capacity was evaluated by placing the dry film specimens (2.0 × 2.0 cm^2^) in a conditioned container at 75% relative humidity (RH) (with a saturated sodium chloride aqueous solution) (Greenspan, 1977) and at room temperature for 2, 6, 24, 48, 72 and 168 h. At each time point, the specimens were weighted (*W*_*w*_), and the moisture absorption was calculated according to the equation:

$$Moisture\;absorption\;\left(\%\right)=\frac{W_{w}-W_{0}}{W_{0}}\times 100$$

where *W*_*0*_ is the initial mass of the dry film. Temperature and environmental air moisture inside the container were monitored using a digital temperature and humidity meter.

#### 2.4.10 Water vapor transmission rate (WVTR) and water vapor permeability (WVP)

WVTR and WVP were determined gravimetrically following the ASTM standard test procedure (ASTM-E96/E96M-10, 2013) for the water method. Briefly, containers (with a diameter of 3.3 cm) were filled with distilled water (100% RH) up to 2 cm of the top, and were sealed with the prepared films (permeation area of 8.55 cm^2^). Then, the film-covered containers were placed in a desiccator with desiccant that had been previously dried at 105 °C for 24 h, to create an atmosphere of 0% RH, at 20 °C. The containers were weighed every hour for 8 h, and five samples of each film were analyzed. All samples were conditioned 24 h prior the test at 23 °C and 50% RH. For comparison, a commercial PVC cling film was also tested. The WVTR and WVP were calculated according to the following equations:

$$WVTR\;\left(gh^{-1}m^{-2}\right)=\frac{G}{t\times A}=\frac{G÷t}{A}$$

$$WVP\;\left(gm^{-1}h^{-1}{Pa}^{-1}\right)=\frac{WVTR}{∆p}=\frac{WVTR}{S\times \left(R_{1}-R_{2}\right)}\times d$$

where *G* is the steady state mass change (g), *t* is the time (h), *G ÷ t* is the slope obtained by linear regression of humidity mass gain, during a transmission time interval (g h^−1^), *A* is the permeation area (m^2^), *Δp* is the vapor pressure difference (Pa), *S* is the saturation vapor pressure at test temperature (Pa), *R*_*1*_ is the relative humidity inside the containers (expressed as a fraction), *R*_*2*_ is the relative humidity inside the desiccator (expressed as a fraction) and *d* is the thickness of the films (m).

### 2.5 *In vitro* antioxidant activity

The *in vitro* antioxidant activity was determined by the DPPH radical scavenging assay (Vilela et al., 2017; Bastante et al., 2021). Briefly, 10 mg of each sample were placed in 4.0 mL of methanol and then 250 µL of a 1 mM DPPH solution were added, and the mixture was kept in the dark at room temperature for 30 min. Afterwards, the absorbance was measured at 517 nm on a Shimadzu UV1800 UV–vis spectrophotometer (Shimadzu Corporation, Kyoto, Japan). The antioxidant activity was calculated based on the following equation:

$$DPPH\;scavenging\;activity\;\left(\%\right)=\frac{A_{control}-A_{sample}}{A_{control}}\times 100$$

where $A_{control}$ corresponds to the absorbance of DPPH in 4.0 mL of methanol and $A_{sample}$ to the absorbance of the sample. The measurements were performed in triplicate.

### 2.6 *In vitro* antibacterial activity

The *in vitro* antibacterial assays followed a procedure based on a previous study with some modifications (Almeida et al., 2023). Firstly, the bacterial strain MRSA DSM 25693 was grown in TSB medium under shaking at 37 °C for 24 h. Then, an aliquot of 2.0 µL was transferred to 30.0 mL of PBS solution to achieve a diluted bacterial suspension with around 10^5^ colony forming units per mL (CFU mL^−1^). Consecutively, 2.0 × 2.0 cm^2^ samples of each film were incubated with 4.0 mL of the PBS suspension in 12-well plates, which was then diluted and plated by the drop plate method (three drops of 10.0 µL for each dilution) onto solid TSA Petri plates. The TSA plates were incubated at 37 °C for 24 h. The embedded films in 12-well plates were incubated at 37 °C for 72 h. Every day, the bacteria concentration (expressed as CFU mL^–1^) was determined by counting the number of bacterial colonies on the most appropriate dilution. This procedure was repeated until reaching 72 h. The bacterial suspension of 10^5^ CFU mL^−1^ was also incubated without any film sample as a negative control. Three independent assays of three replicates were carried out.

### 2.7 Evaluation of the performance of the films on the preservation of a food matrix

The pear fruits were washed with distilled water, peeled, and cut into halves using a stainless-steel knife. Afterwards, each fruit half was cut into roughly cubed shaped pieces (2.0 × 2.0 × 2.0 cm^3^), that were immediately immersed in a sodium hypochlorite aqueous solution during 10 min at 100 ppm for disinfection, followed by immersion in distilled water for another 10 min to remove the sodium hypochlorite. Then, the samples were dried with absorbent paper and placed in aluminum trays coated on the bottom and covered on the top with the prepared films, in sets of 3 cubes of pear per tray. After sealing, each container was stored at +4 °C ± 1 °C for 7 days. For comparison purposes, samples with no film, and with commercial food-grade PVC cling films were also prepared. To assess the performance of the produced films as active packaging systems, the pear samples were analyzed at day 2 and day 7 of storage regarding their physical appearance, browning index (BI), mass loss and pH value.

The physical appearance of the fruit samples was recorded using the Konica Minolta CM-2300d portable sphere type spectrophotometer with horizontal alignment (Konica Minolta Sensing Europe B.V., Warrington, United Kingdom) and the Spectra Magic™ NX software, used to determine the color parameters of the films. The color measurements were obtained at five randomly selected sites of the fruit cubes and performed in triplicate for each aluminum tray. The obtained parameters were also expressed in the CIELab scale and were used to calculate the fruit browning index (BI), according to the equation (Palou et al., 1999):

$$Browning\;index\;\left(BI\right)=\frac{\left[100\left(X-0.31\right)\right]}{0.172}$$

where:

$$X=\frac{\left(a^{*}+{1.75L}^{*}\right)}{\left({5.64L}^{*}+a^{*}-{3.012b}^{*}\right)}$$

The mass loss of the fruits was determined from the difference between the initial mass of each fruit cube ( $W_{i}$ ) and its respective mass at day *n* of the storage ( $W_{n}$ ), using the following equation (Longwei et al., 2021). Measurements were performed in triplicate.

$$Mass\;loss\;\left(\%\right)=\frac{W_{i}-W_{n}}{W_{i}}\times 100$$

The pH of fruit samples was assessed according to the AOAC 981.12 method. Briefly, 10.0 g of fruit were grinded with a hand blender with 100.0 mL of distilled water, before measuring the pH of the solution using a digital benchtop pH meter (pH 50 VioLab benchtop pH meter, XS Instruments, Carpi, Italy). Five measurements were carried out for each sample (Rodríguez et al., 2020).

### 2.8 Statistical analysis

Statistical significance was established at *p* < 0.05 using one-way or two-way variance analysis (ANOVA) and Tukey’s test (GraphPad Prism 8.0, GraphPad Software, San Diego, CA, United States).

## 3 Results and discussion

**FIGURE 1:**
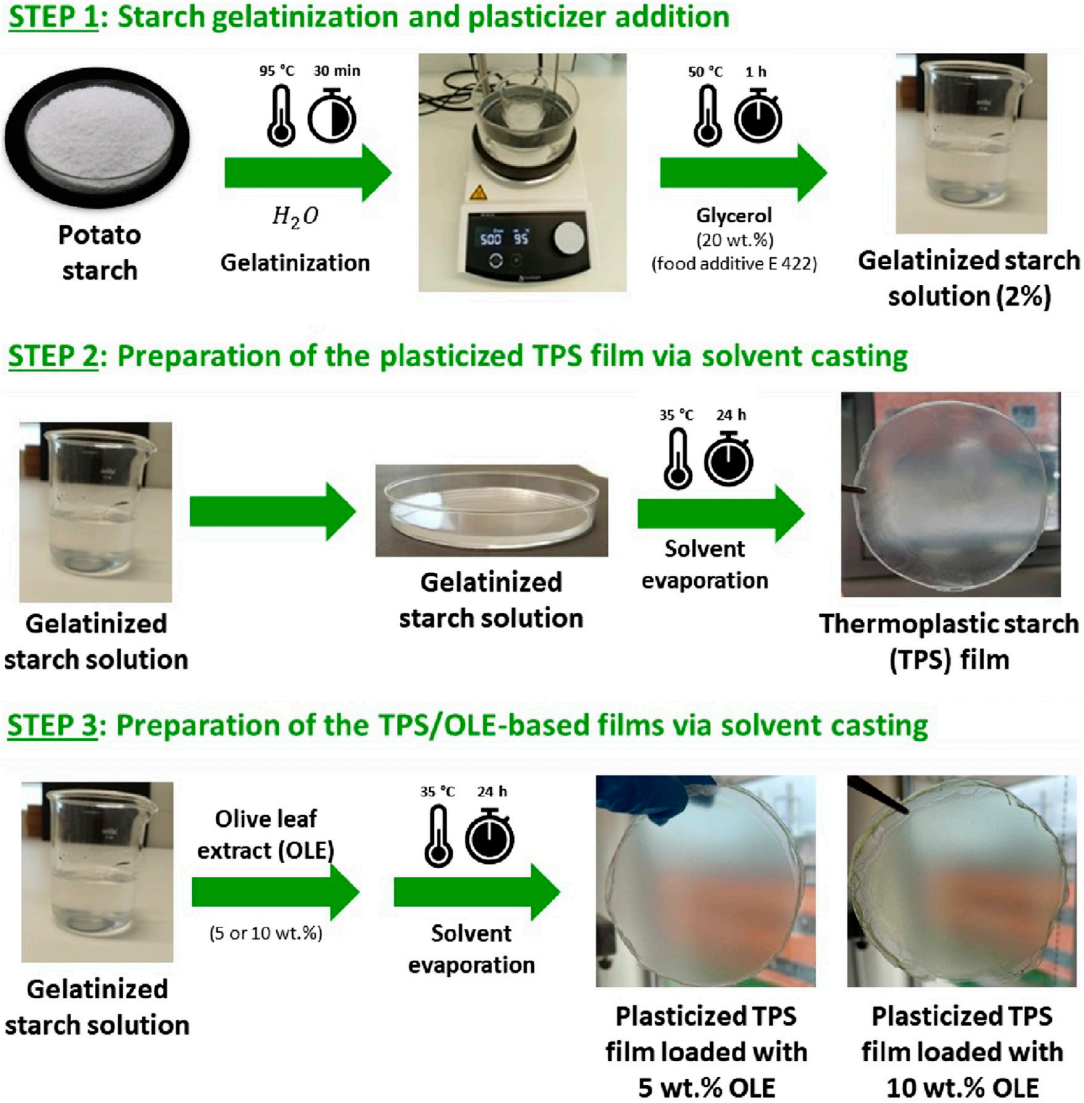
Schematic illustration of the various steps for the preparation of the TPS-based films plasticized with glycerol and loaded with different concentrations of OLE.

Thermoplastic monolayer films composed of TPS and OLE were fabricated via solvent casting, as illustrated in Figure 1. The TPS matrix, classified as generally recognized as safe (GRAS), was selected for its excellent film-forming ability, thermoplastic nature, biodegradability, and suitability for food packaging applications (Khan et al., 2017; Surendren et al., 2022). OLE, extracted via an eco-friendly extraction method (ESE), was incorporated for its potent bioactive properties, particularly its strong antioxidant capacity (EC_50_ = 28.5 ± 1.3 mg L^–1^ (Verano-Naranjo et al., 2025)) and antibacterial activity (e.g., *S*. *aureus*) (Cejudo Bastante et al., 2018; 2019b; 2019a). In addition, a well-known non-toxic and efficient external plasticizer compatible with food applications, namely glycerol (food additive E422), was used to improve flowability, flexibility, and processability of the films (Jha, 2020; Almeida et al., 2023).

Two TPS-based films were prepared using an OLE content of 5.0 and 10.0 wt.% (relative to TPS) and plasticized with glycerol at a content of 20.0 wt.% (relative to TPS), and were compared with the plasticized TPS film without OLE. The OLE and plasticizer contents were elected based on previous studies (Cejudo Bastante et al., 2019b; Almeida et al., 2023; Sarak et al., 2024). Hence, the TPS-based films are composed of 120 or 240 µg of OLE *per* cm^2^ of film (5.0 and 10.0 wt.%, respectively), and 480 µg of plasticizer *per* cm^2^ of film.

The films are visually homogeneous and translucent (Figure 1), and present thickness values of 69 ± 7 μm for TPS, 72 ± 3 µm for TPS_OLE5 and 76 ± 3 µm for TPS_OLE10 (Table 1). Although there appears to be a trend of increasing thickness with higher OLE content, the differences observed are not statistically significant. Similar values were reported after the incorporation of different concentrations of olive fruit extract (García et al., 2020) and Hom Nil rice extract (Sarak et al., 2024) into plasticized TPS films.

The characterization of the TPS-based films in terms of structure (infrared spectroscopy and XRD), morphology (SEM), thermal stability (TGA), mechanical performance (tensile tests), optical properties (color parameters, opacity and UV-vis spectroscopy), water barrier properties (water solubility, moisture absorption, WVTR and WVP), antioxidant activity (DPPH radical scavenging activity), and antibacterial action against a MRSA bacterial strain, is presented below. Furthermore, the performance of the films on the preservation of minimally processed fresh-cut pears was also evaluated during 7 days.

### 3.1 Structural and morphological characterization

**FIGURE 2:**
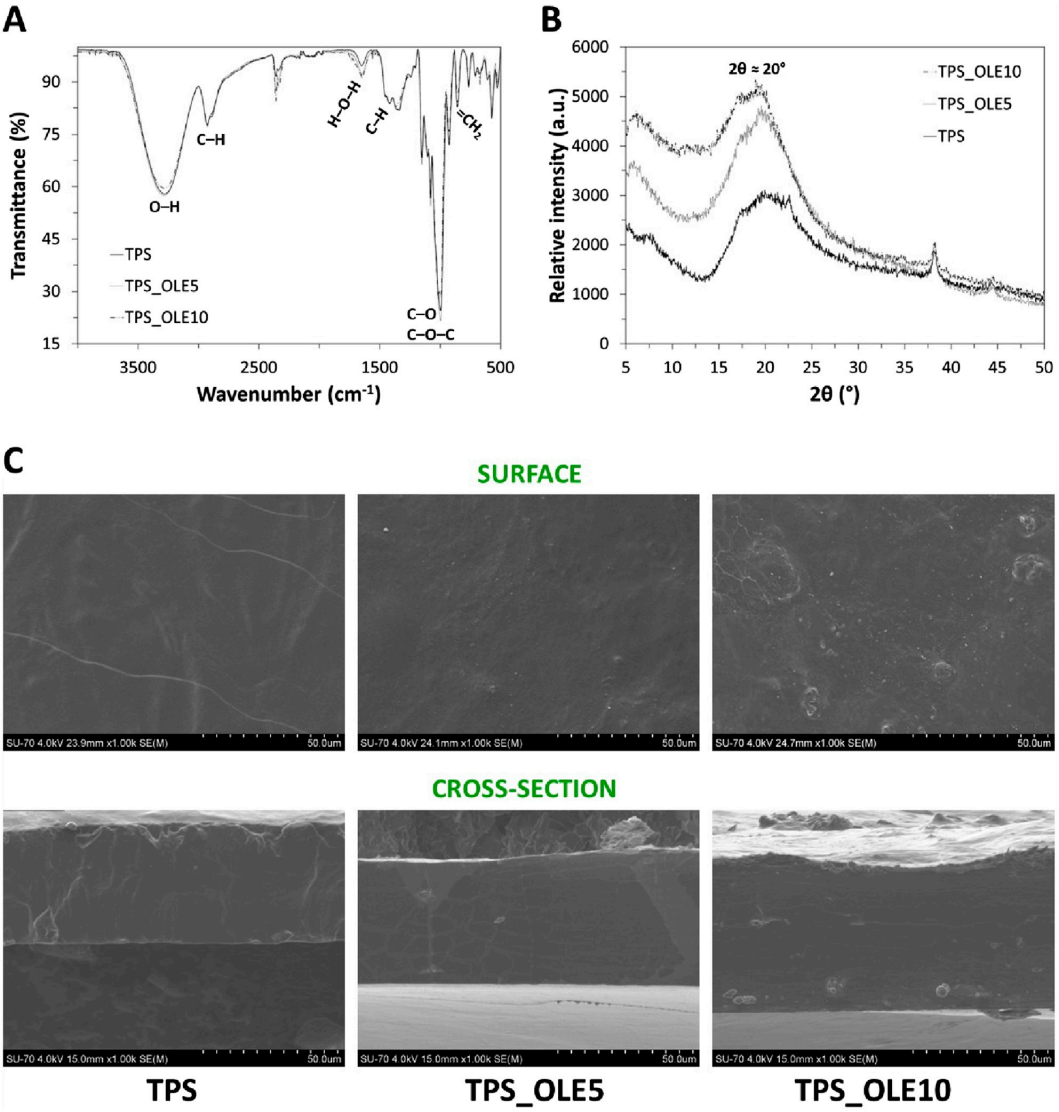
**(A)** ATR-FTIR spectra, **(B)** XRD diffractograms, and **(C)** surface (top) and cross-section (bottom) SEM micrographs of the TPS-based films.

The chemical composition of the TPS-based films was analyzed using infrared spectroscopy, as recorded in Figure 2A. The ATR-FTIR spectrum of the plasticized TPS film displays the characteristic absorption bands of polysaccharides at around 3,280 cm^−1^ (O–H stretching), 2,920 cm^−1^ (C–H stretching), 1,633 cm^–1^ (adsorbed water), 1,415 cm^–1^ (symmetric bending of the C–H groups of–CH_2_OH of the starch chains), and between 1,150 cm^−1^ and 1,000–1,080 cm^−1^ (stretching vibrations of the C–O–C and C–O functional groups, respectively) (Almeida et al., 2023; Khaledian et al., 2024; Sarak et al., 2024). These spectral features are consistent with those observed in literature for plasticized TPS films (García et al., 2020; Almeida et al., 2023).

The ATR-FTIR spectra of the TPS/OLE films are remarkably similar to that of the plasticized TPS (Figure 2A), with no new bands or shifts in peaks position observed. This indicates that the incorporation of the natural extract does not induce any modifications in the structure of the polysaccharide. It is also worth noting that the absorption bands corresponding to various functional groups within the components of the films are likely overlapped, and the relatively low content of additives relative to TPS, primarily OLE (3,300 cm^–1^ (O–H stretching vibration of phenols), 1,086 and 1,046 cm^−1^ (C–OH stretching vibrations in secondary alcohols), and 880 cm^−1^ (=CH_2_ wagging) (Verano-Naranjo et al., 2025), account for the absence of distinct bands typically assigned to these compounds. This trend has been similarly reported in other thermoplastic TPS films loaded with rosemary extract (Piñeros-Hernandez et al., 2017), and Hom Nil rice extract (Sarak et al., 2024) and olive (*Olea europaea* fruit) extract (García et al., 2020).

The TPS-based films were further characterized by XRD, as shown in Figure 2B. The diffraction patterns indicate that the plasticized TPS film has an amorphous structure, characterized by a broad diffraction peak centered at 2θ ≈ 20° (Sarak et al., 2024). This phenomenon is associated with the rearrangement of amylopectin chains during thermo-plasticization of starch, resulting in chain relaxation (Medina Jaramillo et al., 2016; Alikarami et al., 2022). Since the natural extract (OLE) was incorporated at low concentrations, its characteristic diffraction peaks, reported elsewhere (Acosta et al., 2015), were not discernible in the diffractograms of the TPS/OLE-based films. Analogous results were observed in other studies involving TPS films loaded with yerba mate extract (Medina Jaramillo et al., 2016), green tea and basil extracts (Medina-Jaramillo et al., 2020), turnip peel extract (Khaledian et al., 2024) and Hom Nil rice extract (Sarak et al., 2024).

Figure 2C presents a selection of SEM micrographs illustrating the surface and cross-sectional morphology of the TPS-based films. The micrographs reveal good dispersion of OLE and glycerol within the TPS matrix, evidencing their homogeneous distribution and roughly smooth surface and cross-sectional profiles. These observations can be credited to the strong compatibility among the three hydrophilic constituents, namely the polysaccharide, the plasticizer and the natural extract. Similar findings have been reported for bioactive TPS films containing Hom Nil rice extract (2 and 8 wt.%) (Sarak et al., 2024). Notably, the plasticized TPS/OLE films do not exhibit any cracks in their cross-sectional surfaces, contrary to other TPS films plasticized with glycerol (30 wt.%) and loaded with rosemary extract (5, 10% and 20%) (Piñeros-Hernandez et al., 2017).

### 3.2 Thermal and mechanical properties

**FIGURE 3:**
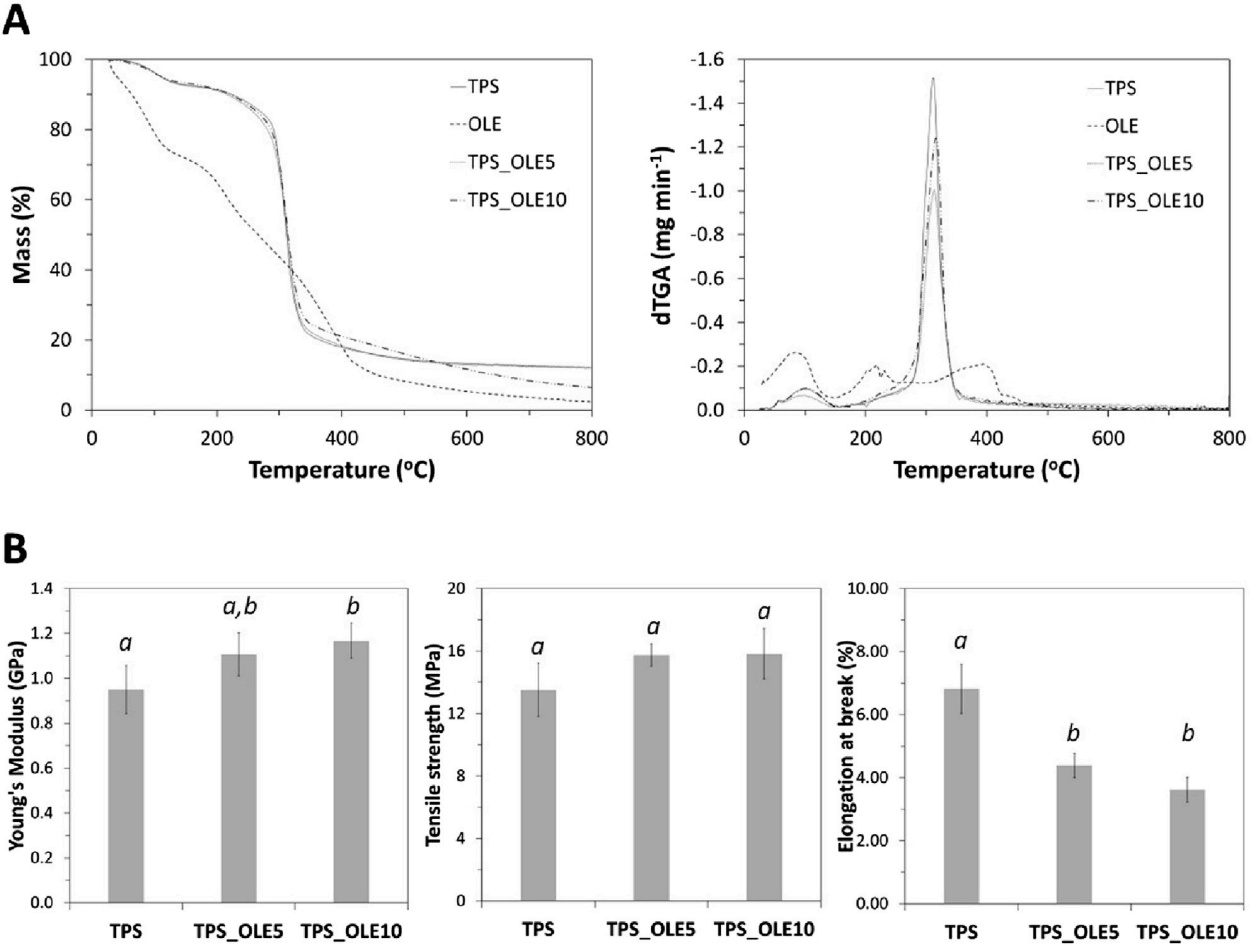
**(A)** Thermograms and the corresponding derivatives of OLE and the plasticized TPS-based films; and **(B)** Young’s modulus, tensile strength, and elongation at break (the values in each graph followed by distinct letters are significantly different (*p* < 0.05)) of the TPS-based films.

Thermogravimetric analysis (TGA) was conducted to assess the thermal stability of TPS-based films under an inert atmosphere, with the resulting data compiled in Figure 3A. The decomposition profile of the natural extract (OLE) aligns with findings reported elsewhere (García et al., 2020).

The TGA tracings of the two TPS/OLE films are analogous to that of the pure plasticized TPS, all exhibiting a single-step degradation profile, aside from the initial mass loss due to the volatilization of water and glycerol. The maximum decomposition temperatures were around 312 °C for TPS, 313 °C for TPS_OLE5 and 315 °C for TPS_OLE10. This means that the incorporation of the OLE into the TPS matrix does not adversely affect the thermal stability of the films. A comparable stability trend has been observed in other TPS-based films containing various phytochemicals (Medina Jaramillo et al., 2016; Piñeros-Hernandez et al., 2017; García et al., 2020; Almeida et al., 2023).

In summary, these results validate that all films are thermally stable up to 200 °C, surpassing the temperatures used in common heat treatments, such as pasteurization (<100 °C) or steam sterilization (110 °C–121 °C) (Almeida et al., 2023). Thus, the films could withstand industrial sterilization processes aimed at eliminating pathogenic microorganisms from food packaged products.

The mechanical properties of the TPS-based films were studied by tensile experiments. The Young’s modulus, tensile strength and elongation at break, determined from the stress-strain curves, are shown in Figure 3B. The plasticized TPS film without the natural extract presented a Young’s modulus of 0.95 ± 0.11 GPa, tensile strength of 13.5 ± 1.7 MPa and elongation at break of 6.8% ± 0.8%, which agrees with previous studies (Almeida et al., 2023). Furthermore, the obtained results demonstrate that the inclusion of OLE led to films that are stiffer and more resistant than the pure plasticized TPS film, as confirmed by the increase in Young’s modulus and tensile strength. Specifically, the Young’s modulus values for the TPS films with 5% and 10% OLE were 1.11 ± 0.10 GPa and 1.17 ± 0.08 GPa (not significantly different), respectively, compared to 0.95 ± 0.11 GPa for the TPS film. The maximum tensile strength values for the TPS films with 5% and 10% OLE were 15.7 ± 0.7 MPa and 15.8 ± 1.6 MPa (not significantly different), respectively, versus 13.5 ± 1.7 MPa for the film without extract. The increase in film stiffness with the extract was accompanied by a decrease in elongation at break. Specifically, the values were 4.4% ± 0.4% and 3.6% ± 0.4% for the films loaded with 5% and 10% OLE, respectively, in comparison to the TPS film without extract, which exhibited a value of 6.8% ± 0.8%. The tensile tests data indicate that the TPS films loaded with this polyphenolic-rich extract are stiffer, yet still remain bendable. Therefore, these TPS-based films are likely well-suited to withstand the mechanical stress encountered during handling, transportation, and storage of food packaging, thereby ensuring durability.

Notably, the studied TPS-based films enriched with OLE exhibited higher Young’s modulus values and comparable tensile strength to commercial 100% biodegradable and compostable food-grade film composed of a blend of PLA, PBAT and a small amount of TPS also loaded with OLE (Verano-Naranjo et al., 2025). When contrasted with other previous studies, the inclusion of extracts of chestnut spiny burs and roasted hazelnut skins also had a minor effect on the mechanical properties of polysaccharide-based films (Esposito et al., 2020).

### 3.3 Optical properties

The coloration of food packaging materials is a critical factor influencing consumer acceptance (Spence and Velasco, 2018). Therefore, the color properties of the TPS-based films were thoroughly examined using spectrophotometric measurements of the CIELab parameters (L*, a*, b*). As presented in Table 2, the data clearly indicate that the addition of OLE significantly influenced the films’ color characteristics, which is also reflected in their visual appearance shown in Figure 1. The incorporation of OLE resulted in an increase in the green (–a*) and yellow (+b*) hue of the films towards greener materials. Additionally, both the lightness (L*) and brightness of the films decreased with the increasing content of OLE compared with the plasticized TPS control, indicating a darker and less luminous appearance. A comparable tendency was recorded for films of TPS loaded with yerba mate extract (Jaramillo et al., 2015) or olive (*Olea europaea* fruit) extract (García et al., 2020), and also PLA/PBAT/TPS films enriched with OLE (Verano-Naranjo et al., 2025).

**TABLE 2:** Colorimetric coordinates, ISO brightness, and opacity of the TPS-based films (The values are expressed as means ± standard deviation; the values in the same column followed by distinct letters are significantly different (*p* < 0.05)).

Film	a*	b*	L*	Brightness	Opacity (mm^–1^)
TPS	1.46 ± 0.14^a^	−0.62 ± 0.13^a^	90.7 ± 0.3^a^	79.8 ± 0.7^a^	1.10 ± 0.07^a^
TPS_OLE5	−2.33 ± 0.27^b^	6.92 ± 0.13^b^	88.9 ± 0.3^b^	64.7 ± 0.7^b^	2.12 ± 0.03^b^
TPS_OLE10	−4.86 ± 0.15^c^	14.30 ± 0.16^c^	85.9 ± 0.5^c^	54.1 ± 0.7^c^	2.27 ± 0.03^c^

Regarding opacity, all TPS-based films exhibited values within the transparency range (<5 (A_600_) mm^–1^), with the lowest opacity observed in the plasticized TPS control film, consistent with previously reported data (Almeida et al., 2023). The incorporation of the bioactive extract, OLE, led to an increase in the opacity of the films, reaching a maximum value of 2.27 ± 0.03 mm^–1^ for TPS_OLE10. This augment was anticipated considering the well-documented light barrier properties of plant secondary metabolites, such as phenolic compounds (Kim and Lee, 2022). These findings align with the visual observations of the TPS-based films depicted in Figure 1. When compared to literature values, the opacity of these films is lower than that of transparent low-density polyethylene (LDPE), which exhibits an opacity of 3.05 (A_600_) mm^–1^ (Shiku et al., 2003), yet higher than the highly transparent poly(vinyl alcohol) (PVA), with an opacity around 1 (A_600_) mm^–1^ (Guzman-Puyol et al., 2022).

Importantly, the greenish hue and relatively low opacity of these TPS-based films are unlikely to hinder their application in food packaging, particularly for light-sensitive food products (Vilela et al., 2018).

**FIGURE 4:**
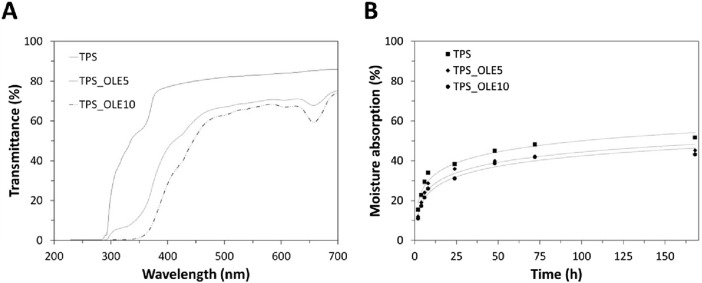
**(A)** Ultraviolet–visible spectra and **(B)** moisture absorption at 75% RH of the TPS-based films.

The light barrier properties of the TPS-based films were evaluated by UV-vis spectroscopy in the range between 200 and 700 nm. As revealed in Figure 4A, the plasticized TPS film is optically translucent, with transmittance values from 77% to 86% in the visible range (between 400 and 700 nm) (Guzman-Puyol et al., 2022), which is in line with the corresponding opacity listed in Table 2.

The films loaded with OLE (i.e., TPS_OLE5 and TPS_OLE10) exhibited reduced transmittance throughout the entire wavelength spectrum (Figure 4A). In the visible range, transmittance values ranged from 46% to 75% for TPS_OLE5 and from 28% to 74% for TPS_OLE10, aligning with the corresponding opacity measurements detailed in Table 2. In the UV range below 315 nm, both plasticized OLE-loaded films have considerably lower transmittance, with values between 0.0%–6.0% for TPS_OLE5 and 0.0%–0.4% for TPS_OLE10. These findings advocate that the films have effective barrier properties to UV radiation, in particular in the UVB region (315–280 nm), and also offer some protection in the UVA range (400–315 nm), as evidenced by transmittance values below 46% for TPS_OLE5 and below 28% for TPS_OLE10. Compared to existing literature, the incorporation of OLE into PLA/PBAT/TPS films originated a comparable behavior with similar transmittance values below 315 nm (Verano-Naranjo et al., 2025). Therefore, the low transmittance observed in TPS/OLE-based films confirm their function as secondary or preventive antioxidants, capable of absorbing UV radiation and thereby partially mitigating the photo-oxidation of light-sensitive food (Vilela et al., 2018; Carvalho et al., 2021).

### 3.4 Water barrier properties

The interaction of the TPS/OLE-based films with water is a relevant property in the context of food packaging, particularly when dealing with fresh food products (e.g., fruits and vegetables). So, the water barrier properties were studied by assessing the films’ water solubility (Table 3), moisture absorption capacity (Figure 4B), water vapor transmission rate (WVTR, Table 3) and water vapor permeability (WVP, Table 3).

**TABLE 3:** Data of water solubility, water vapor transmission rate (WVTR) and water vapor permeability (WVP) of the TPS-based films and the commercial food-grade PVC cling film (the values are expressed as means ± standard deviation; the values in the same column followed by distinct letters are significantly different (*p* < 0.05)).

Film	Water solubility (%)	WVTR (g h^−1^ m^−2^)	WVP (g m^−1^ s^−1^ Pa^−1^)
PVC	–	2.2 ± 0.9^a^	(4.09 ± 1.82)×10^−12a^
TPS	29.5 ± 2.7^a^	14.3 ± 0.4^b^	(1.17 ± 0.03)×10^−10b^
TPS_OLE5	28.7 ± 2.9^a^	15.0 ± 1.1^b,c^	(1.35 ± 0.10)×10^−10b,c^
TPS_OLE10	28.9 ± 2.7^a^	17.4 ± 1.7^c^	(1.48 ± 0.15)×10^−10c^

All TPS-based films were tested for their solubility after 24 h of immersion in water. As summarized in Table 3, the plasticized TPS film showed a solubility of 29.5% ± 2.7%, attributed to the dissolution of starch macromolecular fractions and glycerol, which agrees with values reported in literature for films of this polysaccharide (Almeida et al., 2023; Khaledian et al., 2024). For the TPS/OLE-based films, their water solubility is equivalent to that of plasticized TPS film, with values of 28.7% ± 2.9% for TPS_OLE5 and 28.9% ± 2.7% for TPS_OLE10, indicating that it will be the same fraction of starch and glycerol that is being dissolved. Thus, adding the natural extract (5 and 10 wt.%) did not impact the water solubility of the films. Similar conclusions were obtained for TPS-based films enriched with turnip peel extract (Khaledian et al., 2024) and alginate-based films loaded with OLE (Moura-Alves et al., 2023). Furthermore, the TPS/OLE-based films present water solubility lower than that of Cassava starch based films containing different concentrations of yerba mate extract as antioxidant (0, 5, and 20%) (Jaramillo et al., 2015).

The moisture absorption capacity of the films was assessed by exposing them to a controlled environment with 75% RH at room temperature over a 168 h period, simulating indoor conditions with high humidity. As evidenced in Figure 4B, all films exhibited an initial rapid uptake of moisture, followed by a plateau as they approached equilibrium. No significant differences were observed among the various samples during the initial absorption phase. After 168 h, the moisture absorption percentages were 51.6% ± 6.8%, 45.2% ± 6.8% and 43.1% ± 5.8% for TPS, TPS_OLE5 and TPS_OLE10, respectively. These results demonstrate that increasing the OLE content effectively reduces the hygroscopicity of the TPS films. This decrease in moisture uptake can be explained by the structural organization and strong interactions among the film components, which limit the availability of free hydroxyl groups to interact with water molecules (Jaramillo et al., 2015; Medina Jaramillo et al., 2016). Similar conclusions were obtained for TPS films incorporated with yerba mate extract, supporting the trend of reduced moisture absorption with bioactive compound addition (Medina Jaramillo et al., 2016). The moisture absorption capacity of the TPS/OLE-based films is relevant in the framework of active food packaging, as these films can function as moisture scavengers that help maintain the desired humidity levels, thereby prolonging shelf-life by minimizing condensation inside the package (Yildirim et al., 2018).

The WVTR and WVP were determined for each TPS-based film and compared with food-grade PVC cling film. According to Table 3, the food-grade PVC cling film exhibited a WVTR of 2.2 ± 0.9 g h^−1^ m^−2^, which is lower than the 4.94 g h^−1^ m^−2^ reported for a plasticized PVC film with 12.7 µm of thickness (Steven and Hotchkiss, 2002). Its WVP was (4.09 ± 1.82)×10^−12^ g s^–1^ m^–1^ Pa^−1^, surpassing the value of 2.47 × 10^−13^ g s^–1^ m^–1^ Pa^−1^ reported for a 4 µm thick PVC film (Martiny et al., 2020a).

The plasticized TPS film without extract showed a WVP of (1.17 ± 0.03)×10^−10^ g s^–1^ m^–1^ Pa^−1^, which is lower than values reported for plasticized casava starch films of 200 µm thickness (5.8 × 10^−10^ g s^–1^ m^–1^ Pa^−1^) (Piñeros-Hernandez et al., 2017) and 270 µm thickness (8.8 × 10^−10^ g s^–1^ m^–1^ Pa^−1^) (Jaramillo et al., 2015). These differences are influenced by the testing conditions, and factors such as film thickness, crystallinity and degree of polymerization, which affect the movement of water vapor through the polymer package matrix (González-López et al., 2023).

The addition of OLE to the plasticized TPS films appeared to cause a slight increase in WVP values, although these changes are not statistically significant between both OLE contents. Similar trends have been observed in chitosan films loaded with increasing concentrations of ellagic acid (Vilela et al., 2017). Conversely, the incorporation of yerba mate extract into TPS films reduced their WVP (Jaramillo et al., 2015). Although the WVTR and WVP values of the TPS-based films are higher than those of commercial PVC (Table 3), these films demonstrate significantly lower WVTR values compared to highly permeable materials, such as paper and stretched polypropylene (Hu et al., 2001).

Overall, the fairly low water solubility (Table 3), and moderate moisture absorption capacity (Figure 4B), WVTR and WVP (Table 3) of these TPS-based films represent an advantage within the spectrum of biodegradable active food packaging materials. These films can be suitable not only for dry food products but also for moderately wet foods and fresh food products, highlighting their versatility.

### 3.5 *In vitro* antioxidant and antibacterial activities

**FIGURE 5:**
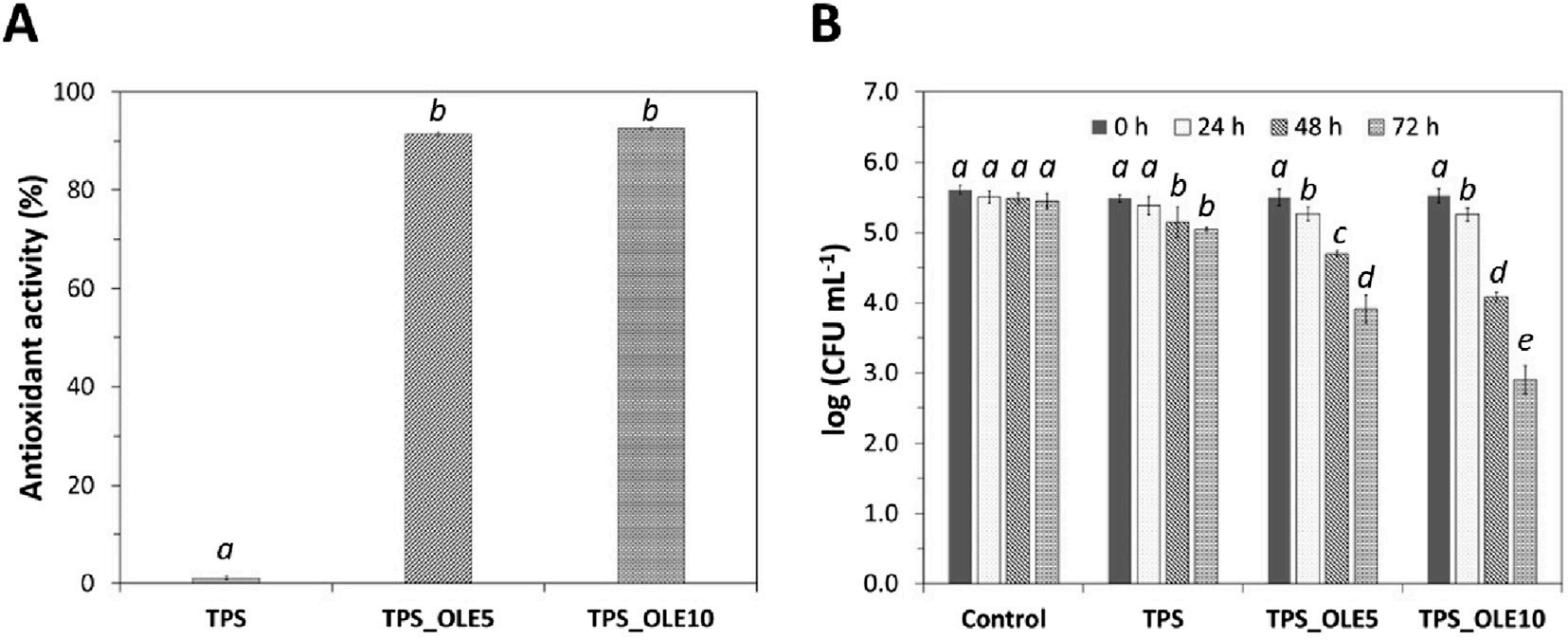
**(A)** Antioxidant activity and **(B)** antibacterial action of the TPS-based films (the values in the graphs followed by distinct letters are significantly different (*p* < 0.05) as determined from the statistical analysis).

OLE is a natural extract with high antioxidant potential due to its high content of phenolic compounds (Grabska-Zielińska, 2024). Therefore, the antioxidant activity of the TPS-based films was determined by the DPPH radical scavenging method. Predictably, the plasticized TPS film has no antioxidant potential with a radical scavenging activity of 1.1% ± 0.3% (Figure 5A), which is in agreement with previous data on this polysaccharide (Almeida et al., 2023), as well as for other thermoplastic materials, such as poly(vinyl alcohol-*co*-ethylene) (Luzi et al., 2018) or poly(lactic acid) (Karamysheva et al., 2024), due to the lack of chemical moieties fitted to act as efficient free-radical scavengers. In contrast, the DPPH radical scavenging activity of the TPS films loaded with OLE reached values of 91.4% ± 0.5% for TPS_OLE5 and 92.6% ± 0.6% for TPS_OLE10 (Figure 5A). These values are comparable to those reported for OLE-loaded PET/PP films (Cejudo Bastante et al., 2017), but higher than those obtained for chitosan films loaded with different concentrations of OLE (10, 15, 20% and 40%) (Famiglietti et al., 2022), TPS films loaded with distinct contents of rosemary extract (5, 10% and 20%) (Piñeros-Hernandez et al., 2017) and TPS films loaded with different concentrations of gallic acid (1.0% and 1.5%) (Almeida et al., 2023).

Based on the data demonstrating antioxidant activity (Figure 5A) and the UV-barrier properties (Figure 4A), the TPS/OLE-based films show great potential as food packaging materials, offering both primary and secondary antioxidant actions, and thus providing an effective strategy to reduce or prevent oxidative degradation in oxidation-sensitive food products (Vilela et al., 2018; Carvalho et al., 2021).

The antibacterial activity of the TPS-based films was assessed against a methicillin-resistant *S*. *aureus* (MRSA) strain. This particular strain was chosen due to its status as a Gram-positive pathogenic bacteria responsible for food spoilage and its frequent involvement in foodborne illnesses (Wendlandt et al., 2013; Sergelidis and Angelidis, 2017). While plasticized TPS alone does not inhibit the growth of MRSA (Almeida et al., 2023), OLE exhibits antibacterial action against *S*. *aureus*, as extensively documented in literature (Cejudo Bastante et al., 2018; Cejudo Bastante et al., 2019b; Cejudo Bastante et al., 2019a), including the antibiotic resistant strain MRSA (Elnahas et al., 2021).

As depicted in Figure 5B, and after enriching the plasticized TPS films with 5% and 10% OLE, a small decrease in the initial bacterial concentration (ca. 0.3-log reduction CFU mL^–1^) was verified after 24 h. After 72 h of contact time, the use of 120 or 240 µg of OLE *per* cm^2^ of film reached a growth inhibition of 1.6- and 2.6-log reduction CFU mL^–1^, respectively. This is a clear indication that the antibacterial action of the TPS/OLE-based films is concentration- and time-dependent, and thus can be tailored. In the context of refrigerated fresh food packaging and fourth-range products, where shelf-life is inherently limited, the extract’s ability to exert a rapid and lasting preservative effect is critical. Although these TPS/OLE-based films with low extract contents have inferior inhibition values against MRSA growth than those reported for TPS films containing a pure phenolic compound, i.e., gallic acid (1.0 and 1.5 wt.%) (Almeida et al., 2023), their antibacterial activity is superior than that of alginate-based films loaded with OLE (Moura-Alves et al., 2023).

According to existing literature, the exact mode of action underlying OLE’s antibacterial activity is not fully understood but it is suggested that its efficacy may be linked to its main constituent, viz. oleuropein. This phenolic compound potentially interferes with the biosynthetic pathways of certain amino acids that are essential for the growth and proliferation of specific microorganisms (Al-Rimawi et al., 2024). Notably, the antibacterial activity of OLE appears to be superior to that of oleuropein alone, suggesting that additional active compounds (e.g., polyphenols and flavonoids) may contribute to or act synergistically with oleuropein (Al-Rimawi et al., 2024). Supporting this, a study on OLE rich in hydroxytyrosol demonstrated antibacterial effects against both reference strains and pathogenic bacteria producing extended-spectrum beta-lactamases (ESBLs) (Ben Hassena et al., 2024). The authors found that hydroxytyrosol could disrupt essential bacterial enzymes crucial for maintaining cell integrity and DNA replication (Ben Hassena et al., 2024), highlighting the potential multifaceted mechanisms through which OLE compounds exert antibacterial activity.

The results obtained for the antibacterial action of the TPS/OLE-based films are highly promising, suggesting that OLE has great potential as an antibacterial additive in active food packaging. Thus, its application could effectively inhibit the growth of pathogenic and spoilage microorganisms responsible for foodborne illnesses and food spoilage, thereby, helping to ensure food safety and quality, and extend shelf-life (Vilela et al., 2018; Carvalho et al., 2021).

### 3.6 Performance of the TPS/OLE films on the preservation of fresh-cut pears

**FIGURE 6:**
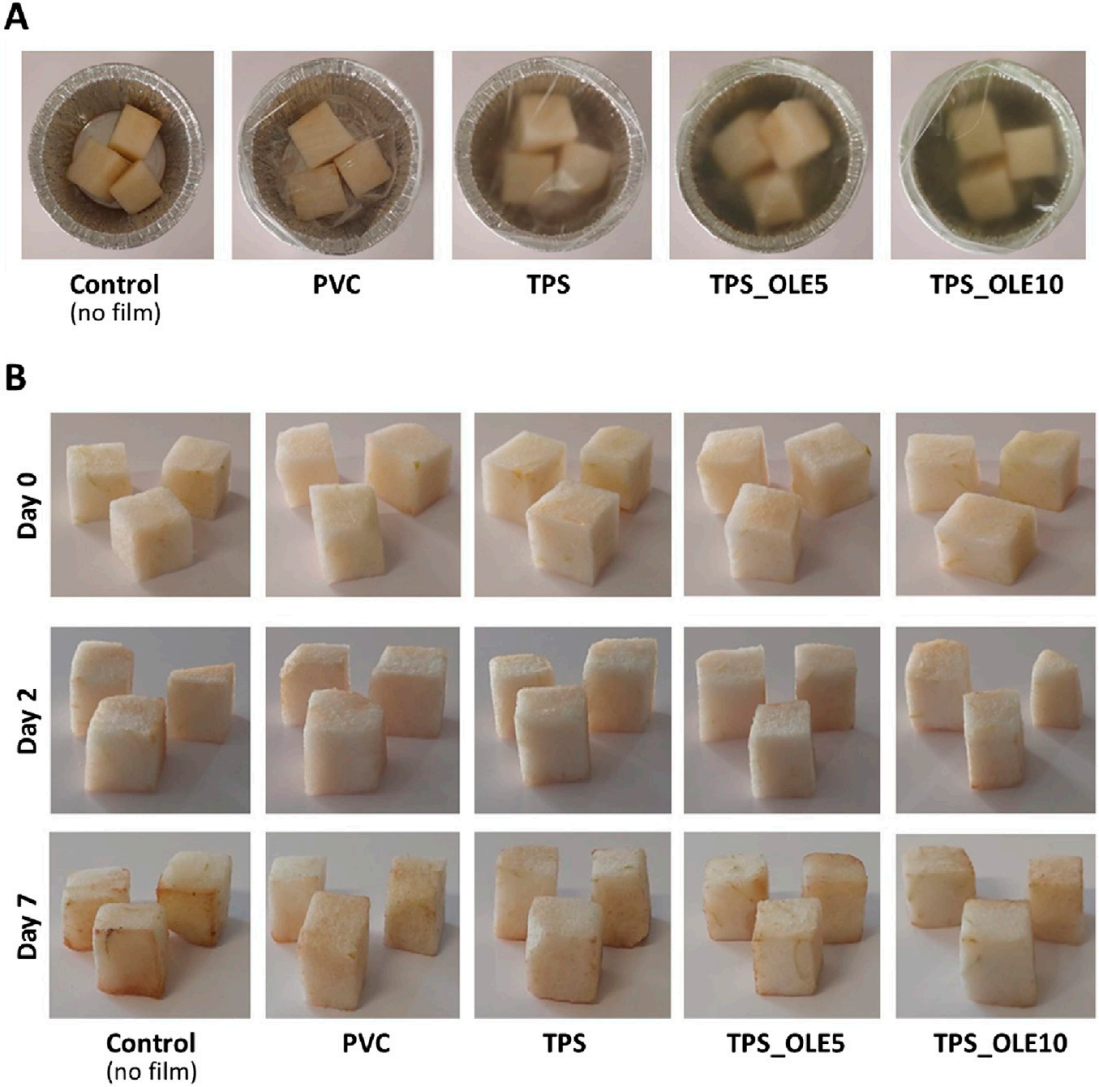
**(A)** Digital photographs of the packed fresh-cut pears with the different films: PVC commercial cling film, plasticized TPS, TPS_OLE5 and TPS_OLE10 at day 0, and **(B)** physical appearance of the fresh-cut pears before and after storage at +4 °C for 2 and 7 days with no film (control) or packed with the different films.

**FIGURE 7:**
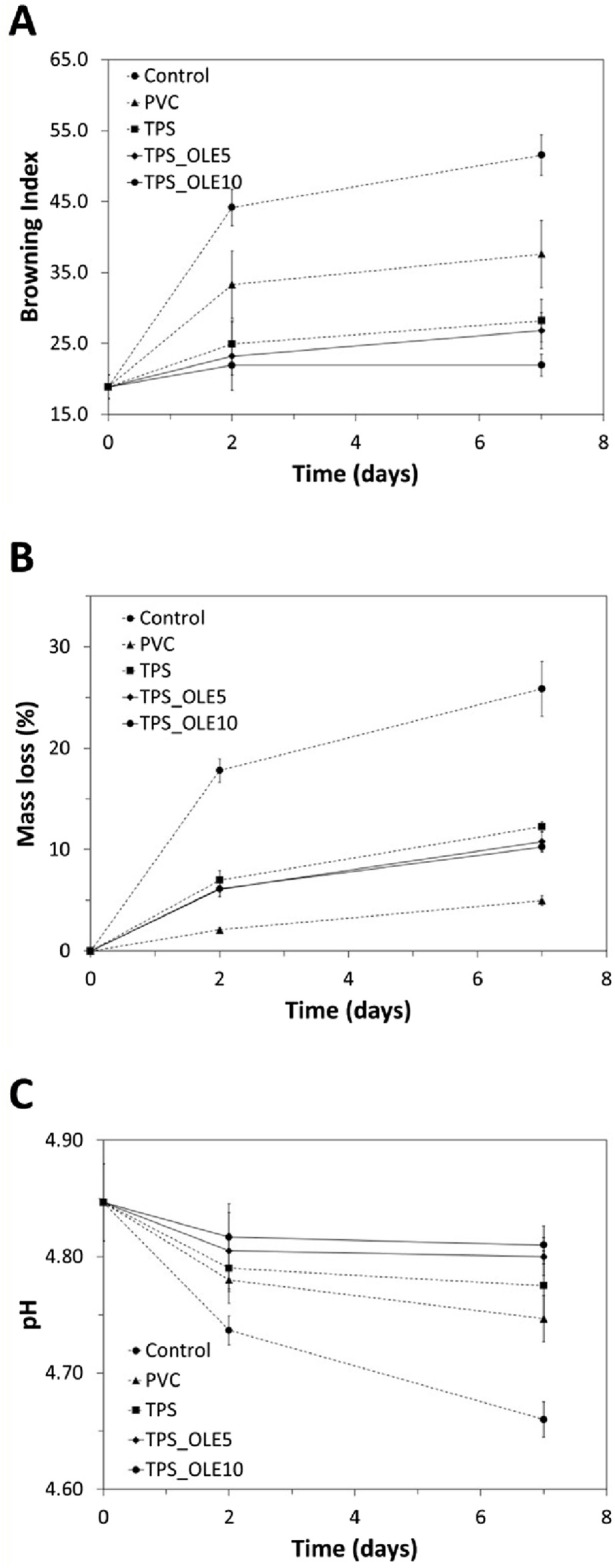
**(A)** Browning index, **(B)** mass loss percentage (%) and **(C)** pH evolution of minimally processed pear samples before and after storage at +4 °C for 2 and 7 days with no film (control) or packed with the different films: food-grade commercial PVC film, TPS, TPS_OLE5 and TPS_OLE 10.

Considering the improved properties the OLE-loaded TPS films, their performance was evaluated in the packaging of a real food matrix and compared with the plasticized TPS film and a commercial food-grade PVC cling film, as illustrated in Figure 6A. To this end, minimally processed fresh-cut pear samples, which are highly prone to oxidation, were packaged using these films. The samples were stored at +4 °C ± 1 °C for 7 days (Figure 6B) and periodically assessed for various quality parameters, including pH, mass loss and browning index (BI) (Figure 7), which are critical indicators of fruit quality and consumer acceptance.

The visual examination of the pear samples over the 7-day storage period (Figure 6B) clearly demonstrates that the OLE-loaded TPS films effectively mitigated browning in the fruit samples as opposed to the control films. This visual observation is corroborated by the BI data presented in Figure 7A. Initially, pear samples had a BI of 18.9 ± 1.7, but, after 7 days at +4 °C ± 1 °C, all samples exhibited increased BI values. However, those packaged with TPS_OLE5 and TPS_OLE10 films showed a notable delay in browning, with BI values of 26.8 ± 2.5 and 21.9 ± 1.6, respectively. In contrast, pears wrapped with commercial PVC film displayed a significantly higher BI of 37.6 ± 4.7. Although the BI for plasticized TPS (28.2 ± 3.0) is not significantly different from that of TPS_OLE5, the difference compared to TPS_OLE10 is considerable. This suggests that the film loaded with 10% OLE offers superior protection against browning, resulting in a BI value that is not statistically different from that of the pears at day zero.

One primary cause of fruit browning is enzymatic oxidation, which is mostly triggered by polyphenol oxidase (PPO), a type-III copper-containing oxidoreductase enzyme present in many fruits and vegetables, including pears (Gomes et al., 2014). Since antioxidants like oleuropein and are known to act as chemical inhibitors of PPO activity (Ortega-García et al., 2008), the antioxidant properties of OLE (Figure 4C) likely play a significant role in reducing browning in fresh-cut pears. Similar effects have been reported by other authors, for instance, packaging fresh-cut pears with wood-inspired biopolymeric nanocomposite films composed of xylans, cellulose nanofibers, and lignosulfonates (Silva et al., 2024), as well as packaging fresh-cut apples with TPS films containing a gallic acid (Almeida et al., 2023).

Regarding the fruit mass loss over time (Figure 7B), the results indicate a progressive increase in mass loss across all samples. After 7 days, pears stored without any protective film experienced the greatest loss (22.6% ± 2.7%), whereas those packaged with food-grade PVC film showed the least loss (5.0% ± 0.5%). The presence of the extract positively influenced this parameter compared to fruit stored with TPS film. Specifically, fresh-cut pears packaged with films containing the extract exhibited mass losses of 11.6% ± 0.9% and 10.3% ± 0.5% after 7 days, corresponding to films with 5% and 10% OLE, respectively. The TPS film alone showed a mass loss of 10.5% ± 0.5%, with no statistically significant difference from the films containing the natural extract. Typically, mass loss during fruit storage results from respiratory activity, moisture transfer and oxidative reactions. Additionally, cutting the pears exposes the fruit without peel to a lower humidity environment, which can lead to substantial dehydration. Comparable results have been observed in other studies, namely with fresh-cut pears packaged with films based on xylans, cellulose nanofibers, and lignosulfonates (Silva et al., 2024), and green peppers wrapped with films formulated from PLA/PBAT/TPS enriched with OLE (Verano-Naranjo et al., 2025).

Finally, Figure 7C depicts the progression of pH in the fresh-cut pears throughout the storage period. Initially, the pears had a pH value of 4.85 ± 0.03, consistent with the findings of other authors (Silva et al., 2024). After 7 days, a decrease in pH was observed in fruit samples stored without a film, reaching 4.66 ± 0.06. In contrast, pears wrapped with the commercial PVC film showed a smaller decline, with a pH of 4.75 ± 0.04. Those packaged with TPS film exhibited a pH of 4.80 ± 0.04, while pears stored with the films containing 5% and 10% extract experienced an even lesser reduction, maintaining pH values of 4.80 ± 0.02 and 4.81 ± 0.02, respectively, indicating effective pH stability over the storage period. Notably, when used to pack sliced pears stored at 4 °C for 7 days, the OLE-enriched films, particularly those with 10% OLE, effectively delayed browning, reduced mass loss and stabilized pH changes.

## 4 Conclusion

Bioactive films composed of thermoplastic starch and an olive leaf extract were successfully fabricated via solvent casting. The ensuing films were visually homogeneous and translucent and exhibited a light-green coloration characteristic of the olive leaf extract. The results obtained for the TPS-based films demonstrate that the inclusion of OLE had a positive effect on the mechanical performance, water resistance and thermal stability of the films. Furthermore, the incorporation of OLE in the plasticized TPS films originated materials with UV-blocking properties and high antioxidant activity, as well as antibacterial action against a MRSA bacterial strain. Finally, when used to pack minimally processed fresh-cut pears stored at +4 °C for 7 days, the OLE-enriched films, particularly those with 10% OLE, effectively delayed browning, mass loss and pH changes. Therefore, these thermoplastic TPS/OLE-based films can potentially be used as active food packaging materials with UV-light protection, antioxidant and antibacterial activities, as an approach to ensure safety, and preserve the quality and extend the shelf-life of fresh food products.

## Data Availability

The original contributions presented in the study are included in the article/supplementary material, further inquiries can be directed to the corresponding author.
